# Mental Health Treatment Should Be an Integrative Part of Caring for Cancer Patients, Caregivers and Families in Japan

**DOI:** 10.31662/jmaj.2018-0008

**Published:** 2018-09-28

**Authors:** Takashi Igarashi

**Affiliations:** 1 National Center for Child Health and Development, Tokyo, Japan

**Keywords:** mental health treatment, depression, anxiety, care for patients, caregivers and families, cancer patients

More than 60 years ago, Brock Chisholm, the first Director-General of the World Health Organization (WHO), stated that “without mental health there can be no true physical health ^(1)^.” We now have significant evidence elucidating the bidirectional relationship between mental disorders and physical health outcomes. Mind and spirit are closely connected to the body. Mental disorders are risk factors that affect the incidence and prognosis of diseases traditionally classified as “noncommunicable,” such as type II diabetes mellitus, chronic obstructive pulmonary disease, and cancer. We cannot address the epidemic of “noncommunicable” diseases without tackling co-morbid mental disorders. Among many noncommunicable diseases, nearly 50% of cancer patients suffer from mental disorders, especially depression and anxiety ^(2)^, and treating symptoms of depression in cancer patients may improve survival time ^(3)^. Improving the immune function positively affects emotions. Cancer has been the leading cause of death since 1981 in Japan, although cancer survival has dramatically improved with the progress in cancer treatment. In this journal, Akechi et al. mentioned the importance of psycho-oncology and showed the essential role of psycho-oncological approach in improving the quality of life in cancer patients and their families ^(4)^.

For patients, caregivers, and families, going through cancer can be a devastating experience. Receiving a fatal diagnosis, going through treatment protocols, and learning to live with limitations can cause depression in many cancer patients, as can side effects from the treatment itself. Treating mental health conditions in cancer patients needs thoughtful management. Because cancer and mental health are deeply personal, it may not be possible to simply encourage a positive attitude, and it may not be possible to remind patients that it will eventually get better. However, it should be kept in mind that treating and caring for the person as a whole, and not just the disease, are beneficial. Mental health conditions are often underdiagnosed in cancer patients, including in children, and a proactive approach must be taken to detect these conditions.

In western countries, cognitive behavioral therapy or dialectical behavioral therapy has been successfully used to treat mental health issues ^(5)^. Treatment strategies include breathing exercises, discussion therapy, laughter therapy, pet therapy, music therapy, behavioral modification, medication for depression or anxiety, or even coping skills development.

Family members of those with cancer are also at increased risk of depression and anxiety. Family members often go through the same grieving process as cancer patients, especially when the cancer is terminal. Caregivers and family members seek support as soon as a diagnosis of cancer is made.

Akechi et al. raised the issues of insufficient number of psycho-oncologists, lack of high-quality evidence concerning the efficacy of prevention, and early detection and treatment of psychological distress experienced by cancer patients and their families in Japan ^(4)^. The oncologist's communications skills training is proven to be effective in preventing psychological distress in cancer patients ^(6)^.

This article sheds light on how cancer impacts mental health and showed that mental health treatment should be an integrative part of caring for cancer patients, caregivers, and families in Japan.

## Article Information

### Conflicts of Interest

None
